# Disease Severity and Cytokine Expression in the Rhinovirus-Induced First Wheezing Episode

**DOI:** 10.3390/v16060924

**Published:** 2024-06-07

**Authors:** Pekka Hurme, Miisa Kähkönen, Beate Rückert, Tero Vahlberg, Riitta Turunen, Tytti Vuorinen, Mübeccel Akdis, Cezmi A. Akdis, Tuomas Jartti

**Affiliations:** 1 Department of Pediatrics and Adolescent Medicine, Turku University Hospital and University of Turku, 20520 Turku, Finland; 2 Swiss Institute of Allergy and Asthma Research (SIAF), University of Zürich, Christine Kühne-Center for Allergy Research and Education (CK-CARE), 7265 Davos, Switzerland; 3 Department of Biostatistics, Turku University Hospital and University of Turku, 20520 Turku, Finland; 4 New Children’s Hospital, Helsinki University Hospital and University of Helsinki, 00290 Helsinki, Finland; 5 Institute of Biomedicine, University of Turku and Turku University Hospital, 20520 Turku, Finland; 6 Department of Clinical Microbiology, Turku University Hospital, 20520 Turku, Finland

**Keywords:** bronchiolitis, cytokine, hospitalization, rhinovirus, virus, wheeze, wheezing

## Abstract

Wheezing children infected with rhinovirus (RV) have a markedly increased risk of subsequently developing recurrencies and asthma. No previous studies have assessed the association between cytokine response and the severity of acute illness in the first wheezing episode in children infected with RV. Forty-seven children treated both as inpatients and as outpatients infected with RV only, aged 3–23 months, with severe first wheezing episodes were recruited. During acute illness, peripheral blood mononuclear cells (PBMCs) were isolated and stimulated with anti-CD3/anti-CD28 in vitro. A multiplex ELISA was used to quantitatively identify 56 different cytokines. The mean age of the children was 17 months, 74% were males, 79% were hospitalized, and 33% were sensitized. In adjusted analyses, the inpatient group was characterized by decreased expressions of interferon gamma (IFN-γ), interleukin 10 (IL-10), macrophage inflammatory protein 1 alpha (MIP-1α), RANTES (CCL5), and tumor necrosis factor-alpha (TNF-α) and an increased expression of ENA-78 (CXCL5) compared to the outpatient group. The cytokine response profiles from the PBMCs were different between the inpatient and outpatient groups. Our results support that firmly controlled interplay between pro-inflammatory and anti-inflammatory responses are required during acute viral infection to absolve the initial infection leading, to less severe illness.

## 1. Introduction

Bronchiolitis is one of the most common illnesses in young children, affecting up to 30% of all children before the age of two years, and it is the most common cause necessitating hospitalization in early childhood [1]. While bronchiolitis is generally considered as a self-limiting condition and the majority of children suffering from bronchiolitis can be treated as outpatients, an estimated 1–2% of all children require hospitalization due to bronchiolitis during their first 24 months of life, and furthermore, up to 10–15% of the overall hospitalizations of children under 24 months are directly linked to bronchiolitis [2]. Additionally, a significant portion of the global mortality in young children, particularly in infants, resulting from an acute respiratory tract infection, is attributed to viral bronchiolitis [2,3,4]. While the general infant mortality rate has decreased, the bronchiolitis-associated rate of mortality has remained relatively stable [2,5]. The risk of severe bronchiolitis has been associated with respiratory syncytial virus (RSV) infection, prematurity, ages under 6 months, exposure to tobacco smoke, congenital heart defects, and Down syndrome [4,5,6]. 

Historically, bronchiolitis has been considered as one condition, leading to a scarcity of data on the morbidity linked with non-RSV viral agents associated with bronchiolitis in children. Though RSV is the predominantly identified viral agent associated with bronchiolitis during the first 12 months of age, rhinovirus (RV) infections become the most prevalent thereafter [1]. Interestingly, emerging evidence is questioning one disease approach, suggesting that bronchiolitis is more of a heterogeneous spectrum encompassing various endotypes that may exhibit different clinical and immunological characteristics as well as clinical long-term prognoses [1]. Moreover, recent studies have indicated that RV-linked bronchiolitis exhibits a stronger association with a subsequent poorer long-term prognosis, including recurrent wheezing and asthma, compared to other causative agents [1,7]. This association is suggested to arise from the interplay between environmental factors (e.g., viruses, allergens) and genetic factors (e.g., immune responses). Hence, children infected with these different bronchiolitis endotypes might benefit from different treatment strategies. In our previous studies, we demonstrated differences in the cytokine responses between wheezing children infected with RV and RSV [8] and observed a decreasing trend in the cytokine responses in young wheezing children infected with both RV and human bocavirus 1 (HBoV1) compared to RV only [9], suggesting that there is a difference in the proposed bronchiolitis endotypes at the cellular level.

The onset of acute wheezing illness in the presence of RV infection induces a swift surge of interferons (type I and III), activating the innate immunity response [1]. Subsequently, cytokines and chemokines are released, leading to various potentially adverse epithelial reactions (cell death, necrosis, sloughing, and unwarranted mucus production). Moreover, variations in the cadherin-related family member 3 (*CDHR3*) gene or 17q21 locus are associated with the severity of the illness and long-term prognosis [1,7,10,11]. Furthermore, RV infections of the airway epithelium, both in human and murine models, have been proposed to induce type 2 innate cytokines, such as IL-25 and IL-33, further enhancing type 2 immunity in the lungs via activating IL-5- and IL-13-producing innate lymphoid cells (ILCs) 2 and T-helper 2 (Th2) cells [12,13,14]. 

There are currently no data on the immunological factors influencing the need for hospitalization or the severity of disease during the first assessment of bronchiolitis in young children with RV-induced wheezing. Therefore, we aimed to compare the cytokine profiles of the children suffering from a severe first wheezing episode caused by RV who were treated as outpatients to those who were treated as inpatients. Our hypothesis is that a difference in cytokine expression from the peripheral blood mononuclear cells (PBMCs) of children with virus-induced acute wheezing is associated with its severity, constituting a more appropriate resolution of the acute disease.

## 2. Materials and Methods

### 2.1. Subjects

The study population was a derivation of the Vinku2 study, which compared the long-term efficacy of oral prednisolone (2 mg/kg/d for 3 days) to a placebo in children with RV-positive first-time wheezing (updated version for seven-year follow-up, trial no. NCT00731575, original version EudraCT 2006-007100-42) [15]. The recruitment of patients was prospectively conducted in the 2007–2010 period at the Department of Pediatrics, Turku University Hospital (Turku, Finland). Patients aged 3–23 months, delivered at ≥36 weeks of gestation, suffering from the first episode of wheezing (parental report and verified from medical charts), and singleton RV infection detected in a nasopharyngeal aspirate (NPA) sample by polymerase chain reaction (PCR) without prior steroid treatment were eligible. The study commenced once written informed consent was provided by a parent or guardian. Children with chronic non-atopic disease, previous corticosteroid treatment, or the need for intensive care were excluded according to the study’s protocol. This study was approved by the Ethics Committee of the Turku University Hospital.

### 2.2. Study Protocol

The requirement for hospitalization was evaluated by an on-duty physician independent of this study. The recruitment of the study subjects was conducted by the study physician. During the enrollment period, the children’s guardians were questioned using a standard survey on risk factors for asthma (host and environmental), and the children were clinically evaluated by the study physician. Furthermore, using a standardized procedure, an NPA sample was taken for viral diagnostics [16], and a blood sample was obtained. After a positive RV-PCR test, the children were randomized to receive either oral prednisolone or a placebo (prednisolone receivers were excluded from the later analyses). The follow-up was conducted as previously described [15]. 

### 2.3. Objective

The objective of this study was to compare the cytokine profiles of admitted and non-admitted children infected with RV who were suffering from severe first-time wheezing.

### 2.4. Definitions

Wheezing was referred to as expiratory breathing difficulty accompanied with bilateral high-pitched whistling sounds. A wheezing event combined by a PCR detection of RV-RNA was called an RV-associated wheezing episode. Atopy was defined as a positive allergen-specific immunoglobulin E (IgE) antibody (cut-off level ≥ 0.35 kU/L) to one or more common allergens (codfish, cow’s milk, egg, peanut, soybean, wheat, cat, dog, horse, birch, mugwort, timothy, *Cladosporium herbarum*, and *Dermatophagoides pteronyssinus*) (Phadiatop Combi^®^, Phadia, Uppsala, Sweden). Aeroallergen sensitization was defined as positive IgE antibodies to one or more of the latter eight allergens, and perennial aeroallergen sensitization was referred to as positive IgE antibodies to dog, cat, or *Dermatophagoides pteronyssinus*. The diagnosis of eczema was based on the characteristic clinical manifestations of the illness, including pruritus, typical morphology, and the chronicity of the condition. If the child exhibited atopic traits (defined above) and presented with eczema, the eczema was categorized as atopic eczema. Type 1 immunity was defined as the activation and function of T-helper 1 cells (Th1), type 1 innate lymphoid cells (ILCs), and classically activated macrophages. Conversely, type 2 immunity was defined as the activation and function of Th2 cells, ILC2s, and macrophages activated by IL-4- and IL-13. Additionally, the involvement of basophils, eosinophils, and mast cells was considered to be part of type 2 immunity. Type 3 immunity was classified as the actions and functions of Th17 cells, ILC3s, and neutrophils. While IFN-γ and IL-12 were referred to as type 1 cytokines, IL-4, IL-5, and IL-13 were referred to as type 2 cytokines, IL-17 and IL-22 were referred to as type 3 cytokines, and IL-10 and TGF-β were referred to as Treg-associated cytokines.

### 2.5. Laboratory Data

A sample of NPA was taken with a nasal swab (nylon flocked dry swab, 520CS01, Copan, Brescia, Italy) and placed at −70 °C. PCR analyses for the detection of viral agents were conducted as previously described [9,16,17,18,19,20]. In short, the extraction of nucleic acids from samples was performed, and in-house reverse transcription (RT)-PCR was used to detect the presence of RV A, B, and C, RSV A and B, and enteroviruses at the Virus Diagnostic Laboratory, Department of Virology, University of Turku (Turku, Finland) [21,22]. Furthermore, the presence of RV A and B, RSV A and B, influenza viruses A and B, human metapneumovirus, parainfluenza virus types 1–3, adenovirus, and coronaviruses 229E, NL63, OC43, and HKU1 was identified with a multiplex PCR test (Seeplex RV12 ACE Detection, Seegene, Seoul, Korea). Additionally, the presence of acute HBoV1 infection was assessed using both PCR and paired sera (IgM and IgG), as described earlier [20,22]. 

The presence of eosinophilia (B-Eos) and the levels of allergen-specific immunoglobulin E (IgE) were assessed according to the standard diagnostics of the Central Laboratory of Turku University Hospital. The quantification of serum 25-hydroxyvitamin D (25(OH)D) levels was performed using liquid chromatography–tandem mass spectrometry at Massachusetts General Hospital (Boston, MA, USA).

During acute illness, peripheral blood was gathered, and PBMCs were extracted using density gradient centrifugation (Ficoll-Paque™ PLUS, GE Healthcare, Amersham, United Kingdom) according to the manufacturer’s instructions. The protocols of processing and storing of PBMCs have previously been described [8]. In brief, samples were stimulated with 0.25 µL of anti-CD3/anti-CD28 (1 μg/mL/1 μg/mL) (BD Biosciences, Franklin Lakes, NJ, USA) for 24 h, and the resulting supernatants were deposited at −80 °C. Following the thawing of the samples, the levels of 56 different cytokines were quantified by using multiplex ELISA with the Bio-Plex 200 System, employing the Bio-Plex Manager 6.0 Software (Bio-Rad, Cressier, Switzerland).

In some samples, during the multiplex analyses, fluorescence failed to reach the quantitative limit of detection (Table S1). Therefore, cytokines that were detected within the limit of quantification in more than half of the samples (29/56, 52%) were included in the further analyses. Samples falling below the limit of detection were set to half the value of the lower threshold of the assay, and samples surpassing the limit of detection were assigned to the upper threshold of the assay [8,9]. The minimum and maximum quantifiable values of the multiplex assays are presented in Tables S2 and S3. 

### 2.6. Statistics

The data distribution was tested using the Kolmogorov–Smirnov test. Due to the skewness of the data, the levels of cytokines were log10- or x^2^-transformed when appropriate. For other statistics, we used the two-sample *t*-test, Mann–Whitney U test, χ^2^ test, or Fisher’s exact test. Furthermore, a multivariable linear model was performed to analyze the differences in cytokine responses between study groups after adjusting for baseline characteristics. The adjustments included baseline characteristics that significantly differed among the study group (sensitization, oxygen saturation at entry, CRP at entry, and parental allergy). For each cytokine, separately, the backward stepwise method was used for the final adjusted model, and only statistically significant baseline characteristic variables were included in the adjusted model (inclusion criteria *p* < 0.05). 

A two-sided *p* < 0.05 was considered statistically significant, and adjustments for multiple testing were not performed due to the nature of this study. Statistical analyses were performed using JMP software (JMP version 13.1.0, SAS Institute, Cary, NC, USA).

## 3. Results

### 3.1. Study Population

**Figure 1:**
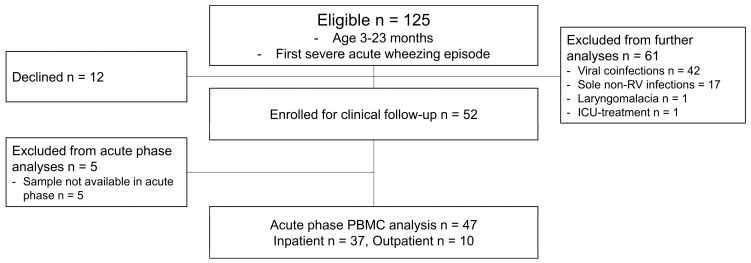
Study flow chart. Patients with cytology data were included. ICU, intensive care unit; PBMC, peripheral blood mononuclear cell; RV, rhinovirus.

At first, 125 children were enrolled in this study, of whom 12 declined to continue. A total of 113 children proceeded with the clinical follow-up. Next, all non-sole RV patients were excluded before further analyses. Thus, 52 children were eligible in the study entry. Subsequently, we excluded 5 children due to the absence of cytology samples, and ultimately, the cytology data of 37 children from the inpatient group and 10 children from the outpatient group were included in the analyses (Figure 1, study flow chart).

### 3.2. Patient Characteristics

The mean age of the patients was 17 months (SD 6), and 74% of the study subjects were males. Moreover, 79% were hospitalized, 33% were atopic, and 22% had atopic eczema. Children who were treated as inpatients had a lower oxygen saturation and higher C-reactive protein (CRP) levels at entry, were more atopic, and were more sensitized to food allergens. Moreover, in the children who were treated as inpatients, the prevalence of the parental allergies were more dominant compared to the children who were treated as outpatients (all *p* < 0.05, Table 1).

### 3.3. Differences in Cytokine Expression at Time of Study Entry 

**Figure 2:**
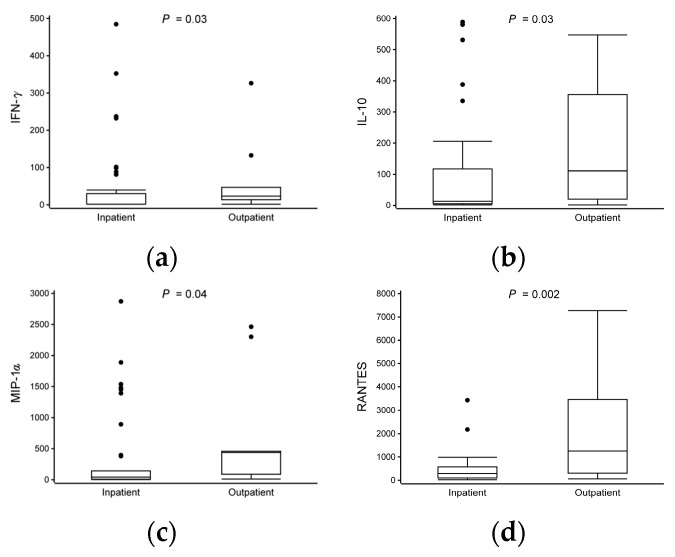

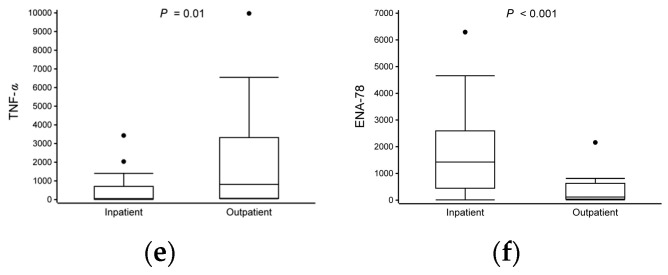
Differences in cytokine expression levels at time of study entry. Data are presented as median and lower (Q1) and upper (Q3) quartiles, and data falling outside Q1–Q3 range are plotted as outliers. In IFN-γ, for better visualization, one sample from both study groups were excluded from figure but included in analyses [inpatient (5200 pg/mL) and outpatient (2900 pg/mL)]. Cytokine concentrations are presented as pg/mL. Multiple significant differences in cytokine expression were observed between study groups (inpatient vs. outpatient, all *p* < 0.05) (**a**–**f**).

After stimulation with anti-CD3 and anti-CD28, we observed significant differences in the cytokine responses between the patients treated as inpatients and those treated as outpatients. The hospitalized group had lower expressions of IFN-γ (median 1.6 vs. 24 pg/mL), IL-10 (13 vs. 110), MIP-1α (42 vs. 440), RANTES (290 vs. 1300), and TNF-α (52 vs. 810) and a higher expression of ENA-78 (1400 vs. 120) compared to the non-hospitalized group (all *p* < 0.05, Table 2, Figure 2, Table S2). The expressions of IL-1β and IL-6 were marked but not statistically significant (all *p* < 0.09, Table 2 and Table S4).

**Table 2:** Differences in cytokine expression levels at time of study entry.

Cytokine	Inpatient n = 37	Outpatient n = 10	*p*-Value Univariate	*p*-Value Multivariate	Adjustments
IFN-γ	1.6 (1.5–60)	24 (18–180)	**0.02**	**0.03**	1
IL-10	13 (5.0–120)	110 (18–380)	0.09	**0.03**	1
IL-1β	1.6 (1.6–15)	80 (1.6–1200)	0.10	0.07	-
IL-6	25 (5.5–270)	1000 (7.4–2200)	0.12	0.09	-
MIP-1α	42 (8.5–260)	440 (69–920)	**0.03**	**0.04**	-
RANTES	290 (97–590)	1300 (290–3600)	**0.005**	**0.002**	-
TNF-α	52 (16–820)	810 (60–4100)	**0.01**	**0.01**	-
Eotaxin-2	780 (680–1100)	670 (420–810)	0.054	0.31	3
ENA-78	1400 (410–2700)	120 (36–670)	**0.003**	**<0.001**	-

Values are presented as medians (interquartile range). Data were analyzed using Mann–Whitney U-test and multivariable linear model. Log_10_-transformed cytokine expression levels were used in analyses. Adjustments for immunologic analyses included baseline characteristics that significantly differed between groups (sensitization = 1, oxygen saturation at entry = 2, CRP at entry = 3, parental allergy = 4). A backward stepwise method was used for the final adjustment model separately for each cytokine. Only statistically significant baseline characteristic variables (*p* < 0.05) were included in the final model. All data are presented in Supplementary Table S4. Bold text; statistical significance at *p <* 0.05.

## 4. Discussion

While the overall cytokine response in both the outpatient and inpatient groups exhibited similarities, importantly, the inpatient group displayed a more pronounced downward trend across both pro- and anti-inflammatory cytokine profiles. Distinctions between the different subtypes of cell-mediated immunity have been described in detail before [23]. Notably, the hospitalized children were characterized by decreased expressions of IFN-γ, IL-10, MIP-1α, RANTES, and TNF-α and an increased expression of ENA-78 compared to the non-hospitalized children. 

Classically, both IFN-γ and TNF-α are considered pro-inflammatory cytokines. The former is considered as a central effector of the immune system, specifically associated with type 1 immunity, and it is known for its involvement in both host defense and immune surveillance, as well as immune-regulatory capabilities countering viral infections via the inhibition of viral replication [24]. The latter is associated with both type 1 and type 3 immune responses and is known for its crucial role in host defense in response to infections and injuries, in the induction of proinflammatory proteins, as well as in the promotion of the differentiation of Th17 cells [25]. Additionally, IFN-γ is a major cytotoxic T-cell proliferator [26]. 

MIP-1α is primarily a macrophage-derived chemokine known for its proinflammatory capabilities and the chemotaxis of eosinophils, macrophages, and lymphocytes [27]. Interestingly, MIP-1α, as well as RANTES, is also associated with the proliferation of airway smooth muscle, leading to airway tissue remodeling [28,29]. Moreover, during bronchiolitis, the increased expression of MIP-1α has been associated with the severity of illness, a longer duration of supplemental oxygen therapy [30,31], and recurrent wheezing [32]. However, the latter was only observed in hospitalized children, and comparisons were not made with non-hospitalized children.

RANTES, a known chemoattractant of eosinophils, basophils, monocytes, and lymphocytes, is commonly recognized as a type 2 inflammation-associated chemokine [33,34]. However, in recent studies, it has also been suggested to have a more pleiotropic role in cell-mediated immunity. Furthermore, in a recent study, a decreased expression of RANTES from PBMCs after RV infection was observed in children with asthma compared to non-asthmatic children [35]. This finding is in line with our results. ENA-78 is primarily known for the chemotaxis of neutrophil, particularly during the early stages of infection [36], and moreover, ENA-78 is indirectly involved in B-cell chemotaxis via CXCL13 [37]. 

Interestingly, the children who were treated as outpatients showed a more profound anti-inflammatory cytokine response characterized by increased levels of IL-10, which is associated with Treg responses, compared to those who were treated as inpatients. IL-10 is crucial in downregulating the inflammatory effects of type 1–3 inflammation. However, the data concerning the effects of IL-10 in bronchiolitis are contradicting. Though increased levels of IL-10 have been reported to be associated with recurrent wheezing [38], and lower IL-10 levels have been shown to be associated with more severe diseases [39], conversely, IL-10 has not been reported to play a role in the severity of disease [40]. Interestingly, a study focusing on IL-10 over-expression in a transgenic mouse model indicated that IL-10 had a dualistic effect regarding the severity of illness in RSV infection (attenuating in the acute phase, but in later phases, the effect might be additive), suggesting that the levels and the effect of IL-10 are time-dependent [41]. This finding is in line with our results. In addition, a prior study observing the cytokine responses from ILC2s showed diminished cytokine levels of IFN-γ and IL-10 in children with recurrent wheezing compared to children with viral bronchiolitis [42]. This observation aligns with our own findings. 

Surprisingly, the non-hospitalized children were also characterized by more of a marked pro-inflammatory response compared to the hospitalized children, as demonstrated by the higher expressions of IFN-γ, TNF-α, and MIP-1α. This may indicate that both closely controlled pro- and anti-inflammatory responses are required to adequately absolve the initial infection, leading to less severe illness. Importantly, the dysregulation of Treg cells has been associated with more profound type 2-skewed immune responses, contributing to asthma development [43]. 

In our previous studies, we demonstrated that the cytokine profiles of children infected with RV and RSV differ from each other and that HBoV1, while occurring as a coinfection with RV, decreases the overall cytokine response in the first severe wheezing episode [8,9]. Thus, this emphasizes the possibility of different bronchiolitis endotypes. This study is in line with these results. RV-associated wheezing illness, at least partly, shares both clinical and pathophysiological asthma-like characteristics [1]. While the cytokine expression in both study groups generally resembled each other, the children who were treated as outpatients appeared to express a more robust and more controlled cytokine response overall (pro- and anti-inflammatory) compared to those who were treated as inpatients.

While the activation of PBMCs via anti-CD3/anti-CD28 closely resembles the natural activation of T-cells via the T-cell receptor [44], variations in stimulation protocols can induce modifications in cytokine response profiles. Consequently, a comparison of our results to those from other studies differing in stimulation protocols presents a challenge, and our findings should be validated under comparable conditions. Intriguingly, certain significantly different cytokine responses were not derived from T-cells. Conversely, the activation of PBMCs under the presence of anti-CD3/anti-CD28 may lead to the indirect activation of other types of lymphocytes, leading to a heightened expression of non-T-cell-derived cytokines [45]. The cytokine assays used in our study were broad and capable of detecting various inflammatory responses beyond those originating solely from T-cells. This was partly intentional, given the novelty of our study design and protocol, making it difficult to predict specific cytokine differences or responses.

The current study is fortified by numerous strengths, including the careful selection of study participants, thorough viral diagnostics, and in-depth analyses of cytokine response profiles. The original hypothesis of our current study aimed to differentiate cytokine responses between the hospitalized and non-hospitalized children and, therefore, the absence of a “control” group. Nevertheless, our study also exhibits some limitations. Firstly, while an a priori power calculation was carried out to test our primary hypothesis, there was no power calculation for this analytic design, and the moderately limited number of study subjects prevented the use of ideal multivariable model analyses. Nonetheless, both study groups originated from a novel bronchiolitis subgroup. Secondly, the restricted volume of culture medium posed a challenge in the execution of the dilution series, leading to the fluorescence of several cytokines to exceed the upper limit of quantification, consequently complicating the analyses of these cytokines. Nevertheless, the quantity of impacted cytokines was modest (Table S1). Thirdly, although different RV serotypes may exhibit distinct behaviors and trigger the immune system through different mechanisms, the cases in our study were mainly RVA- or RVC-positive with no RVB infections identified. However, RVA and RVC are both associated with more severe disease, which may explain the lack of differences between the serotypes. Fourthly, flow cytometry was not performed. Therefore, there is a possibility that the differences in cytokine responses may, at least partly, reflect the differences in the proportion of PBMCs that are T-cells between the study groups. However, contrary to this, the differences were not universal across all cytokines, and in 80% of the analyses, no significant difference was found between the study groups, indicating that the expression of those cytokines was equal. Further, the cytokine responses observed from the PBMCs stimulated with anti-CD3/anti-CD28 may vary from the responses in other regions of the body, such as in the respiratory tract. Finally, despite the fact that early wheezing disease in children infected with RV has been more strongly associated with risks of both subsequent recurrencies and asthma compared to other viral agents [7], due to the restricted sample size, the long-term prognoses of the study groups could not be assessed.

In summary, our current study supports the emerging assumption that bronchiolitis is a spectrum encompassing different endotypes. Our findings also support that robust and controlled cytokine and chemokine responses are required to avoid the need for hospitalization. Our study also reveals potential new biomarkers for early events of asthma in high-risk cohorts, namely first-time wheezing children infected with RV. However, further trials are warranted.

## Figures and Tables

**Table 1:** Patient characteristics at time of study entry.

Characteristic	Inpatient (n = 37)	Outpatient (n = 10)	*p*-Value
Age, months	13.3 (5.7)	12.4 (5.4)	0.63
Male sex, no.	29 (78%)	6 (60%)	0.25
Weight, kg	10.5 (2.1)	10.6 (2.1)	0.85
Preceding wheezing, days	1 (1–1)	1 (1–2)	0.11
Preceding cough, days	2 (1–3)	2 (3–5)	0.08
Preceding rhinitis, days	3 (2–5)	4 (3–6)	0.06
Preceding temperature over 37.5 °C	1 (0–2)	0 (0–2)	0.48
Clinical score, points	6 (4–9)	5 (4–7)	0.39
Oxygen saturation, %	96 (95–98)	98 (97–100)	**0.02**
Temperature, °C	37.5 (0.6)	37.2 (0.5)	0.24
CRP, mg/L	15 (7–23)	7 (2–16)	**0.048**
B-Eos (1 × 10^9^/L)	0.53 (0.35–0.84) *	0.48 (0.31–0.61) *	0.42
B-Eos > 0.4 × 10^9^/L	24 (67%) *	5 (56%) *	0.70
Total IgE	26 (11–56)	12 (6–24) *	0.06
Eczema, no.	11 (30%)	0 (0%)	0.09
Atopic eczema, no.	11 (30%)	0 (0%)	0.09
Sensitization, no.	15 (41%)	0 (0%) *	**0.02**
Food, no.	13 (35%)	0 (0%) *	**0.04**
Aero, no.	10 (27%)	0 (0%) *	0.17
Perennial, no.	9 (24%)	0 (0%) *	0.17
Parental asthma, no.	7 (19%)	2 (20%)	1.0
Parental allergy, no.	27 (73%)	2 (20%)	**0.004**
Parental smoking, no.	16 (43%)	6 (60%)	0.48
S-25-OHD, nmol/L	5150 (770–18,200)	2000 (1.6–59,750)	0.42

B-Eos, blood eosinophil count; S-25-OHD, serum 25-hydroxyvitamin D. Values are presented as mean (standard deviation), median (interquartile range), or number (%). Data were analyzed using two-sample *t*-test, Mann–Whitney U-test, χ^2^ test, or Fisher’s exact test. Bold text; statistical significance *p* < 0.05. * One sample missing due to absence of blood sample.

## Data Availability

The raw data supporting the conclusions of this article will be made available by the authors on request.

## Supplementary Materials

The following supporting information can be downloaded at https://www.mdpi.com/article/10.3390/v16060924/s1, Table S1: The quantification of the cytokines; Table S2: Minimum limits of quantification of cytokine plates; Table S3: Maximum limits of quantification of cytokine plates; Table S4: Differences in cytokine expression levels at time of study entry.

## Abbreviations

ENA-78	epithelial-derived neutrophil-activating peptide 78, CXCL5
IFN-γ	interferon gamma
IL-1β	interleukin 1 beta
IL-10	interleukin 10
MIP-1α	macrophage inflammatory protein-1 alpha
PBMC	peripheral blood mononuclear cell
RANTES	Regulated upon activation, normal T-cell expressed and presumably secreted, CCL5
TNF-α	tumor necrosis factor-alpha
RV	rhinovirus
