# Supplementary material for: Disease Severity and Cytokine Expression in the Rhinovirus-Induced First Wheezing Episode
Source: Viruses. 2024 Jun 7;16(6):924. doi: 10.3390/v16060924 (PMC11209381; doi:10.3390/v16060924)
Supplement: Supplementary file 1 [file viruses-16-00924-s001.zip › viruses-3008700-supplementary.pdf]

Disease severity and cytokine expression in the rhinovirus-induced first wheezing episode

Pekka Hurme,[1] Miisa Kähkönen,[1] Beate Rückert,[2] Tero Vahlberg,[3] Riitta Turunen,[1,4] Tytti Vuorinen,[5,6] Mübeccel Akdis,[2] Cezmi A. Akdis,[2] Tuomas Jartti[1]

[1]Department of Pediatrics and Adolescent Medicine, Turku University Hospital and University of Turku, 20520, Turku, Finland.
[2]Swiss Institute of Allergy and Asthma Research (SIAF), University of Zürich, Christine Kühne-Center for Allergy Research and Education (CK-CARE), 7265, Davos, Switzerland.
[3]Department of Biostatistics, University of Turku, 20520, Turku, Finland
[4]New Children's Hospital, Helsinki University Hospital and University of Helsinki, 00290, Helsinki, Finland
[5]Institute of Biomedicine, University of Turku and Turku University Hospital, 20520, Turku, Finland.
[6]Department of Clinical Microbiology, Turku University Hospital, 20520, Turku, Finland.

Table S1. The quantification of the cytokines

Cytokine	Acute phase n=97					Convalescent phase n=91				
	Within range	OOR <	OOR >	*	***	Within range	OOR <	OOR >	*	***
EGF	55 (57%)	17		25		32 (35%)	21		38	
FGF-2	39 (40%)	57		1		26 (29%)	65			
Eotaxin	12 (12%)	46		39		11 (125)	36		44	
TGF-α	14 (14%)	63		20		12 (13%)	59		20	
G-CSF	75 (77%)	20		1		64 (70%)	25		2	
GM-CSF	53 (55%)	10		23	11	45 (49%)	20		15	10
Fractalkine	71 (73%)	23		1	2	72 (79%)	19			
IFN-α2	73 (75%)	9		14	1	47 (52%)	26		18	
IFN-γ	57 (59%)	12		28		52 (57%)	19		20	
IL-10	85 (88%)	1		11		75 (82%)	3		13	
MCP-3	71 (73%)	1	25			60 (66%)	1	27	3	
IL-12P40	31 (32%)	53		13		31 (34%)	50		10	
MDC	91 (94%)		2	4		87 (96%)	1	1	2	
IL-12P70	26 (27%)	25		46		27 (30%)	27		37	
IL-13	64 (66%)	23		10		58 (64%)	24		9	
IL-15	3 (3%)	77		17		5 (5%)	73		13	
sCD40L	42 (43%)	36		19		35 (38%)	40		16	
IL-17A	34 (35%)	59		4		22 (24%)	61		8	
IL-1RA	95 (98%)	1		1		85 (93%)	2		4	
IL-1α	36 (37%)	40		21		37 (41%)	42		12	
IL-9	6 (6%)	62	1	28		6 (7%)	57		28	
IL-1β	53 (55%)	10		34		46 (51%)	12		32	
IL-2	51 (53%)	21		25		46 (51%)	30		15	
IL-3	15 (15%)	45		37		10 (11%)	46		35	
IL-4	42 (43%)	44		11		38 (42%)	47		6	
IL-5	22 (23%)	32		43		18 (20%)	28		45	
IL-6	72 (74%)	10	7	7	1	51 (56%)	16	7	17	
IL-7	43 (44%)	37		15	2	33 (36%)	44		14	
IL-8	91 (94%)		5	1		86 (95%)		3	2	
IP-10	71 (73%)	1	16	9		73 (80%)		11	7	
MCP-1	71 (73%)		11	15		69 (76%)		5	17	
MIP-1α	79 (81%)	1	5	12		71 (78%)		4	16	
MIP-1β	97 (100%)					91 (100%)				
RANTES	97 (100%)					90 (99%)			1	
TNF-α	94 (97%)			3		81 (89%)			10	
VEGF	54 (56%)	32		11		45 (49%)	32		14	
Eotaxin-2	97 (100%)					89 (98%)	1		1	
MCP-2	87 (90%)		2	8		84 (92%)			7	
MCP-4	46 (47%)	2		45	4	30 (33%)	7		53	1
I-309	93 (96%)	4				87 (96%)	4			
IL-16	95 (98%)				2	89 (98%)	1		1	
TARC	86 (89%)			8	3	82 (90%)			8	1
Eotaxin-3	53 (55%)	44				45 (49%)	46			
LIF	4 (4%)	33		54	6	7 (8%)	45		37	2
TPO	5 (5%)	49		42	1	5 (5%)	48		38	
SCF	3 (3%)	68		26		3 (3%)	66		22	
TSLP	2 (2%)	54		39	2	1 (1%)	53		37	
IL-33	1 (1%)	51		44	1	1 (1%)	53		37	
IL-20	38 (39%)	28		29	2	37 (41%)	29		24	1
IL-21	1 (1%)	70		23	3	1 (1%)	66		21	3
IL-23	20 (21%)	51		26		16 (18%)	48		27	
TRAIL	3 (3%)	30		64		5 (5%)	35		51	
SDF-1α+β	35 (36%)	41		21		32 (35%)	35		24	
ENA-78	91 (94%)	1		5		85 (93%)	4		2	
MIP1-d	6 (6%)	60		29	2	8 (9%)	59		23	1
IL-28A	3 (3%)	82		12		4 (4%)	78		9	

Values are shown as number (%).
OOR< = fluorescence under the detection limit; OOR> = fluorescence exceeds of detection limit; * = sample value extrapolated; *** = sample missing
Cytokine concentrations measured by multiplex ELISA using fluorescence from PBMC culture medium

Table S2. Minimum limits of quantification of the cytokine plates

Cytokine plate (LLOQ)	Median	IQR
EGF	3.20	3.20-3.21
FGF-2	20.7	15.6-80.0
Eotaxin	3.25	3.23-3.28
TGF-α	3.20	3.19-3.20
G-CSF	17.7	3.96-47.6
GM-CSF	3.19	3.05-3.23
Fractalkine	16.5	13.3-18.4
IFN-α2	2.92	2.72-3.08
IFN-γ	3.22	3.19-3.26
IL-10	3.20	3.19-3.21
MCP-3	3.36	3.28-3.48
IL-12P40	3.13	3.08-3.17
MDC	3.23	3.21-3.28
IL-12P70	3.24	3.21-3.25
IL-13	2.90	2.76-3.08
IL-15	3.10	3.03-3.13
sCD40L	2.84	2.67-3.10
IL-17A	2.82	2.63-3.03
IL-1RA	2.99	2.90-3.13
IL-1α	3.21	3.19-3.24
IL-9	3.20	3.18-3.22
IL-1β	3.20	3.17-3.22
IL-2	3.18	3.16-3.25
IL-3	3.20	3.19-3.22
IL-4	3.97	2.57-17.9
IL-5	3.22	3.12-3.23
IL-6	3.17	3.16-3.22
IL-7	2.96	2.87-3.17
IL-8	3.19	3.17-3.20
IP-10	15.3	13.9-19.5
MCP-1	3.22	3.21-3.24
MIP-1α	3.12	3.09-3.18
MIP-1β	3.15	2.84-17.3
RANTES	3.02	2.88-3.13
TNF-α	3.20	3.16-3.24
VEGF	422	47.8-1510
Eotaxin-2	9.78	9.73-9.84
MCP-2	4.88	4.86-4.89
MCP-4	9.66	9.62-9.75
I-309	8.55	2.23-9.23
IL-16	9.44	8.58-10.1
TARC	0.977	0.974-0.979
Eotaxin-3	227	116-253
LIF	19.5	19.2-19.7
TPO	48.5	47.8-49.6
SCF	9.62	9.53-9.87
TSLP	9.78	9.77-9.79
IL-33	19.5	19.4-19.6
IL-20	48.8	47.9-49.7
IL-21	19.5	19.5-19.5
IL-23	47.2	44.3-49.0
TRAIL	9.72	9.56-9.77
SDF-1α+β	96.2	95.2-98.0
ENA-78	19.5	19.4-19.6
MIP1-d	48.8	48.8-48.9
IL-28A	9.32	8.56-9.72

Values shown as median (pg/ml), and IQR.
LLOQ, lower limit of quantification; IQR, interquartile range.
All Elisa cytokines analyze plates, and their lower limits of detection and plate variation are included.

Table S3. Maximum limits of quantification of the cytokine plates

Cytokine plate (ULOQ)	Median (pg/ml)	IRQ (pq/ml)
EGF	2380	1020-8360
FGF-2	10000	9960-10100
Eotaxin	378	371-383
TGF-α	452	422-1530
G-CSF	10000	10000-10000
GM-CSF	10000	9940-10100
Fractalkine	10000	9960-10100
IFN-α2	10000	10000-10000
IFN-γ	10000	9980-10000
IL-10	10000	9990-10000
MCP-3	391	384-395
IL-12P40	10200	10100-10200
MDC	9310	8670-9580
IL-12P70	10200	10100-10300
IL-13	10000	10000-10000
IL-15	10100	10100-10200
sCD40L	10000	7840-10000
IL-17A	10200	10100-10300
IL-1RA	10100	10000-10100
IL-1α	9940	9710-10100
IL-9	9740	8500-10100
IL-1β	9960	9400-10500
IL-2	10100	9680-10300
IL-3	9920	9770-10100
IL-4	10000	10000-10000
IL-5	8090	2230-9270
IL-6	1960	1850-5090
IL-7	1960	1910-1980
IL-8	8500	1830-9580
IP-10	10000	9860-10400
MCP-1	8330	4950-8970
MIP-1α	439	408-2150
MIP-1β	10400	10200-11100
RANTES	10400	5180-11000
TNF-α	9970	9540-10600
VEGF	10900	10300-11700
Eotaxin-2	1860	619-2450
MCP-2	1440	306-4110
MCP-4	1870	652-3050
I-309	2000	1020-2010
IL-16	9390	2260-11500
TARC	941	825-1060
Eotaxin-3	50000	37300-50000
LIF	20000	19800-20600
TPO	50100	49700-51400
SCF	10100	9990-10100
TSLP	9880	9430-10600
IL-33	15100	4260-18800
IL-20	48900	45200-54600
IL-21	19000	4160-22100
IL-23	50000	50000-50300
TRAIL	9910	9690-10100
SDF-1α+β	97800	25700-110000
ENA-78	14500	4150-17800
MIP1-d	3600	3140-9290
IL-28A	10000	10000-10000

Values shown as median (pg/ml), and IQR.
ULOQ, upper limit of quantification; IQR, interquartile range.
All Elisa cytokines analyze plates, and their upper limits of detection and plate variation are included.

Detailed version of the PBMC extraction protocol

Blood samples for peripheral blood mononuclear cell (PBMC) processing (>8 ml) were collected to LH Lithium Heparin tube in two different time points (at the study entry and at the convalescent phase [2-week follow-up]). Both samples were analyzed with identical fashion and using the same procedure. After the blood sample was collected, the samples were placed in a rocking shaker and were slowly rocked in a room temperature until the beginning of PBMC processing, which was performed on the same day.

At the beginning of PBMC processing, the sample was centrifuged at 1200 rpm for 10 minutes in +22°C. Thereafter, the plasma was extracted to 15ml falcon tube and frozen. After the plasma extraction, the remaining cell pellet was moved to 50 ml falcon tube after which a phosphate-buffered saline (PBS) solution was added on the top of the cell pellet (final volume of the mixture 20 ml). The sample-PBS-mixture was then carefully divided into two new 15ml falcon tubes each containing 3 ml of room temperature Ficoll-Plaque™ PLUS (GE Healthcare, Amersham, United Kingdom). The mixture was then centrifuged at 2200 rpm for 22 minutes in +22°C. All the visible PBS was then extracted with pipette and PBMCs from both 15 ml falcon tubes was extracted with Pasteur pipette to a new 50 ml falcon tube. 30 ml of PBS was then added on the top of the PBMCs, and the mixture then centrifuged at 1200 rpm for 10 minutes in +22°C. All visible PBS was then extracted, and solution was diluted with 1 ml of PBS. 10 µl of PBMC-PBS-mixture and 90 µl of TryptanBlue (0.5%) was then mixed in Eppendorf tube, and the cells were placed in a Bürker chamber, and the cells were calculated.

After cell calculation, mixture was then diluted that the final sample would contain 2 million cells per ml. Adequately diluted sample was then carefully divided to a 48-well culture plate (one stimulant per well) containing the stimulants. Each well contained 0.5x10^6 PBMCs (in volume of 250 µl) and stimulant (25 µl) diluted to 225 µl of medium (total volume was 500 µl/well). PBMCs were stimulated with anti-CD3/anti-CD28 ([20 µg/ml / 20 µg/ml; final 1 µg/ml / 1 µg/ml; i.e. 0.5 µg/well] BD Biosciences, Franklin Lakes, NJ, USA) in 5% CO2 incubator for 24h and after that the cell and the medium mixture were collected to 2 ml Eppendorf tubes. The samples were centrifuged in 1200 rpm, 1 min at room temperature after which the supernatants were collected and stored in a -80°C refrigerator.

Later, the supernatants were shipped inside dry ice containers to the Swiss Institute of Allergy and Asthma Research (SIAF), Davos, Switzerland. Upon arrival the samples were still frozen and stored in -80°C refrigerator until analysis. Samples were defrosted right before the analyses and analyzed with Millipore HCYTOMAG-60K-36 and HCYP2MAG-62K-20 assay

(Merck KGaA, Darmstadt, Germany) using the Bio-Plex 200 System utilizing the Bio-Plex Manager 6.0 Software (Bio-Rad, Cressier, Switzerland) to perform profiling of 56 different cytokines (Table S1-3). Internal quality controls for all analytes were satisfactory.

Stimulants

More than 700 vials of anti-CD3/anti-CD28 20µg/ml /20µg/ml (final 1 µg/ml / 1 µg/ml), i.e. 0.5 µg/well (BD Biosciences, Franklin Lakes, NJ, USA) were frozen before study (300 to be used at acute phase, 300 at convalescent phase and 100 for reserve). The stimulants were prepared prior to the study at the same time to ensure that concentration of the stimulants remained stable throughout the study period. Each stimulant was be frozen in 25 µl aliquots in –80 °C and defrosted in room temperature just before use in PBMC cultures (0.5 x 10^6 cells/well, final volume 0.5 ml).

Medium

Medium contained 100 ml RPMI-16400, 10 ml iFBS, 1 ml L-glutamine, 20 µl gentamicin, 2 ml HEPES.

Cytokine analyses

Due to the limitations of quantitative multiplex Elisa profiling, a few cytokines did not reach the quantitative limit of detection (i.e., fluorescence was under or exceeded the quantification limit of the assay) (Table S3). These values were identified as lower limit of quantification (LLOQ) and upper limit of quantification (ULOQ). Each cytokine found in more than 50% of patient samples within the limit of quantification (values between LLOQ and ULOQ) were included for analysis, thus ensuring that conclusions would not be based on minority of samples (Table S1-2). Sample was classified as out of range when signal, i.e., fluorescence fell out of the assay range which was both precise and accurate. Due to aforementioned, of 56 cytokines 29 were later classified as eligible for statistical analyses. Samples under the limit of detection were assigned half the value of the LLOQ (Table S1), and samples exceeding the upper limit of detection (either reported as "ULOQ" or a numerical extrapolated value greater than the assays upper limit of quantification), the values were set to the ULOQ threshold of the assay (Table S2).

Table S4. Differences in cytokine expression levels at study entry

**Cytokine**	**Inpatient n=37**	**Outpatient n=10**	**p-value univariate**	**p-value multivariate**	**Adjustments**
EGF	3.4 (1.6-4.2)	4.2 (1.6-5.0)	0.57	0.95	-
G-CSF	8.9 (8.0-18)	51 (6.5-530)	0.26	0.13	-
GM-CSF	8.3 (1.6-90)	17 (8.2-380)	0.11	0.19	-
Fractalkine	15 (8.3-28)	9.5 (7.8-23)	0.27	0.36	-
IFN-α2	4.7 (3.0-22)	44 (4.4-140)	0.13	0.21	-
IFN-γ	1.6 (1.5-60)	24 (18-180)	**0.02**	**0.03**	1
IL-10	13 (5.0-120)	110 (18-380)	0.09	**0.03**	1
MCP-3	340 (110-390)	390 (110-390)	0.45	0.95	-
MDC	300 (130-1800)	200 (110-1300)	0.48	0.77	-
IL-13	5.5 (1.5-17)	5.6 (1.5-75)	0.79	0.77	-
IL-1RA	97 (24-480)	82 (40-210)	0.64	0.59	1
IL-1β	1.6 (1.6-15)	80 (1.6-1200)	0.10	0.07	-
IL-2	3.4 (1.6-22)	8.4 (1.6-22)	0.89	0.83	-
IL-6	25 (5.5-270)	1000 (7.4-2200)	0.12	0.09	-
IL-8	1600 (650-2600)	780 (620-1700)	0.19	0.30	-
IP-10	1200 (170-10000)	2900 (390-8100)	0.50	0.11	1
MCP-1	6600 (4100-8300)	7900 (4900-8300)	0.48	0.78	2
MIP-1α	42 (8.5-260)	440 (69-920)	**0.03**	**0.04**	-
MIP-1β	210 (48-870)	590 (190-1000)	0.15	0.12	-
RANTES	290 (97-590)	1300 (290-3600)	**0.005**	**0.002**	-
TNF-α	52 (16-820)	810 (60-4100)	**0.01**	**0.01**	-
VEGF	59 (13-210)	210 (8.9-210)	0.74	0.71	-
Eotaxin-2	780 (680-1100)	670 (420-810)	0.054	0.31	3
MCP-2	610 (210-1300)	470 (73-1000)	0.40	0.38	2
I-309	32 (20-65)	28 (12-85)	0.98	0.82	-
IL-16	70 (51-91)	56 (36-110)	0.38	0.73	-
TARC	4.0 (2.1-9.1)	2.7 (1.4-8.4)	0.61	0.62	-
Eotaxin-3	150 (110-220)	110 (110-210)	0.69	0.50	-
ENA-78	1400 (410-2700)	120 (36-670)	**0.003**	**< 0.001**	-

Values are presented as medians (interquartile range). Data were analyzed using Mann–Whitney U-test and multivariable linear model. Log10-transformed cytokine expression levels were used in analyses. Adjustments for immunologic analyses included baseline characteristics that significantly differed between groups (sensitization = 1, oxygen saturation at entry = 2, CRP at entry = 3, parental allergy = 4). A backward stepwise method was used for the final adjustment model separately for each cytokine. Only statistically significant baseline characteristic variables ($p < 0.05$) were included in the final model. Bold text; statistical significance at $p < 0.05$.
